# Improving the utilization of essential public health services by Chinese elderly migrants: strategies and policy implication

**DOI:** 10.7189/jogh.10.010807

**Published:** 2020-06

**Authors:** Shangfeng Tang, Chengxu Long, Ruoxi Wang, Qiaoyan Liu, Da Feng, Zhanchun Feng

**Affiliations:** 1 School of Medicine and Health Management, Tongji Medical College, Huazhong University of Science and Technology, Wuhan, Hubei, China; 2 Research Institute of Rehabilitation Information, China Rehabilitation Science Institute, Beijing, China; 3 School of Pharmacy, Tongji Medical College, Huazhong University of Science and Technology, Wuhan, Hubei, China

## Abstract

**Background:**

The concept of healthy aging has become a global health strategy in response to the population aging. In China, old-aged migrants are facing serious health care challenges due to the obstacles in the utilization of health services, social integration and ignored public policies. We aimed to examine the old-aged migrants’ utilization of the essential public health services and its underlying factors on account of change of residence, and social support.

**Methods:**

Data came from the senior sample (aged over 65 years, n = 11 161) of the 2015 National Migrant Dynamic Monitoring Survey in China that employed Probability Proportionate to Size method as a sampling strategy. χ^2^ tests and binary multilevel model were conducted to analyze the difference and the underlying factors of the utilization of essential health services among old-aged migrants.

**Results:**

Approximately 66.2% of old-aged migrants did not receive free physician examination services from health institutions in the past year, and 34.6% of old-aged migrants with chronic disease have been followed up by doctors. There were significant differences in the utilization of essential public health services among old-aged migrants across different individuals and families. It showed that exercise time, migrating range, migrating reason, physical health condition, chronic disease, local friends, health insurance, household expenditure, and income were significantly associated with the elderly migrants’ utilization of essential public health services.

**Conclusions:**

The utilization of essential public health services among old-aged migrants was insufficient in comparison with the general population. The government should launch targeted policies such as production and work-related injuries for the floating population. The supply side should promote the equalization of essential public health services for migrants. Social organizations and community should undertake the responsibility in social support for old-aged migrants.

Population aging has become a major problem facing human society. It is predicted that by 2050, the elderly over 60 years will account for 33% of the population in developed countries and 19% of those in developing countries [1]. China's population aging is also becoming a serious problem. By the end of 2018, those aged 60 years and above accounted for 17.9% of the general population, whereas the proportion of people over 65 years old was 11.9%in China [2]. The elderly in this study refer to the domestic migrants who have migrated from their original residence to another domestic immigration area for more than one month but have not registered, with aged over 65 years old. Employment, caring for the younger generation, and living after retirement constituted the three main reasons for the migration of the senior [3]. In 2015, the number of old-aged migrants over the age of 65 in China reached 8.18 million [3], accounting for 5.7% of the total population of the senior [4].

The utilization of essential public health services (EPHS) among old-aged migrants is another challenging issue [5,6], and migration status in the household registration system is the core factor hindering the utilization of public services [7,8]. Meanwhile, as the previous study reported that weak health literacy and economic status contributes to insufficient understanding or utilization of health insurance and EPHS [9]. Besides, social network reconstruction and conservative awareness make it difficult for old-aged migrants adapting to the local community in a short time [10]. Of course, it attracts the authorities’ attention. As the report of the 19th National Congress of the Communist Party of China pointed out that it is urgent to actively respond to the population aging and build better social environment. The “Healthy China 2030 Strategy” calls for integration of elderly care and medical services and enhancing the health monitoring and comprehensive intervention for geriatric conditions and chronic diseases. Since 2003, China sequentially carried out comprehensive management policy for the floating population. After 2010, the focus shifted from management to service and paid more attention to service equalization. For instance, China promulgated the Pilot Work of Basic Public Services Equalization for Floating Population in 2013. How to improve the old-aged migrants’ utilization of EPHS has become an important practical issue that should not be ignored in the context of the growth of old-aged migrants.

However, there are limited studies both on the supply side and demand side to delivery EPHS for old-aged migrants. The most current studies focus on working-age migrants, while the associated factors with the utilization among the working-age population may not apply to illustrate those of old-aged migrants [11,12]. Although prior studies explored various association factors with the health service’ seeking behavior, mainly focusing on the individual level [13]. The influence of family, social support and migration characteristics of old-aged migrants was explored insufficient when compared to those among the general populations. Simultaneously, it is well-known that the previous studies consistently focused on disease treatment attracted the most attention in the public and academic community, while few of them paid attention to preventive care for the migrants [14].

According to the determinant health model, the utilization of health services is an essential determinant of health status. How to benefit the health, focusing on the old-aged migrants’ improvement of EPHS utilization is a vital question. In hence, on the background of the advanced aging and urbanization in China, this study aims to discover the disparities in the utilization of EPHS among old-aged migrants with different characteristics and to explore the impact of social support, family floating, health factors on the utilization of EPHS for old-aged migrants. Moreover, it will provide implications for international researchers and policymakers beyond the single national context. The essential public health services, including the physical examinations for the elderly and follow-up services for chronic disease patients, are universal and inclusive public services which are available in different countries around the world. Nevertheless, it is noteworthy that the migrants in this study just refer to the population migrated in different regions among the country, which is different from the conditions among the international migration. Therefore, caution is needed when we extend the implication to the international population migration.

## METHODS

### Selection and description of participants

The data in 2015 China Migrant Dynamic Monitoring Survey we used was second-hand data that was applied from national health commissions of China. This was a national survey organized by the Chinese Migrant Population Service Centre. The survey adopted a stratified, multi-stage, scale-oriented Probability Proportionate to Size method as a sampling strategy. Taking the data of the annual report of the entire floating population in 32 provincial-level units as the basic sampling frame, covering approximately 10 000 sample points. The details of data collection procedures were available at the website of http://www.chinaldrk.org.cn/wjw/#/application/index as a registered member. In the process, the respondents signed informed consent and used a paper-and-pencil self-management questionnaire, which was anonymous and confidential throughout the process.

The survey is mainly targeted for migrants over 15 years old, while the last survey specifically for old-aged migrants was only carried out in 2015. To investigate the old-aged migrants’ physical health condition and their public health service utilization, the sample of old-aged migrants in the volume A of 2015 Migrant Dynamic Monitoring Survey was selected to conduct the data analysis. Due to the community free annually physical examination was only covered the elderly over 65 years of age, migrants over 65 years old (born before May 1950) were included as a sample of community physical examinations (N = 11161) in this cross-sectional study. To use the samples sufficiently, the whole investigated elderly over 60 years of age (born before May 1955) were included in the analysis of followed up services utilization among elderly patients with chronic conditions (N = 2898).

### Measurement

**Table 1** presents the multilevel variables and assignments. Two types of old-aged migrants’ utilization of EPHS was measured by two dependent variables, including physical examinations (whether received community health stations or center’ annually free physical examinations), and follow-up services (whether received chronic follow-up services by doctors), which were accordingly coded as Yes (1) and No (0).

**Table 1:** Variables and assignments

Variables	Assignments
Utilization of essential public health services:	
Community annually free physical examinations	0 = no; 1 = yes
Chronic disease follow-up services	0 = no; 1 = yes
Demographic and behavioral factors	
Age(years)	1 = 60-65; 2 = 65-70; 3 = 70-80; 4 = >80
Ethnicity	1 = Han; 2 = Else
Daily exercise time (minutes)	1 = <30; 2 = 30-60; 3 = >60
Floating factors:	
Floating range	1 = cross-provincial; 2 = cross-city; 3 = cross-county; 4 = cross-border
Floating reason	1 = employment; 2 = caring children; 3 = treatment; 4 = retirement; 5 = else
Health factors:	
Health status	1 = health; 2 = general health; 3 = unhealth but self-care; 4 = no self-care
Chronic disease	1 = yes; 2 = no
Social support factors:	
Numbers of local friends	1 = 0; 2 = 1-5; 3 = >5
Health insurance	1 = yes; 2 = no
Family factors:	
Household monthly expenditure	1 = <¥2000; 2 = ¥2000-5000; 3 = >¥5000
Household monthly income after tax	1 = <¥5000; 2 = ¥5000-10000; 3 = >¥10000

Considering data sample and literature research [15], the independent variables included five layers of data on sociodemographic, migration, health, social support and family. The following were included in the study based on the availability in the data set and theoretical relationship with the dependent and explanatory variables: Age groups; Ethnicity groups; Daily exercise time; Floating range; Floating reason; Health status; With or without chronic disease; Numbers of local friends; Health insurance; Household monthly expenditure; Household monthly income after tax. The details of the independent variables were showed in **Table 1**.

### Statistics

The study employed descriptive statistics, χ^2^ test and binary logistic regression of the multilevel model were employed in this study. The frequency and percentage characteristics of old-aged migrants were described first. Then, the χ^2^ test was conducted to summarize the difference in the utilization of EPHS among those with different characteristics through SPSS21.0 (IBM, Almond, New York, USA) statistical software.

Besides, the binary logistic multilevel regression model was employed to explore the impact of different factors on the senior migrants’ utilization of EPHS. Multilevel model was applied to analyze the hierarchical structure data, in which each lower-level unit clustered within one higher-level unit. The dependent variables were binary responses, so binary logistic regression of the multilevel model was conducted. There was a two-level data structure including individuals (level 1) nested within different families (level 2). Level 1 presented the potential in individual-level, such as sociodemographic, migration, physical health condition, and social support. Level 2 represented family settings, such as household expenditure and household income. Above all, the empty model test was conducted only with initial service utilization and residual. The Intra-Class Correlation (ICC) = intercept/ (residual + intercept) was calculated in random effects analysis. The higher the ICC, the more prominent structural features of the data, which suggests the necessity to conduct multilevel model analysis. Then, sociodemographic, migration, physical health condition, social support factors were simultaneously added to the model. After that, household economic factors were added to make a fully adjusted model finally. Each test employed intercept (initial service utilization) and residual as random effects. Based on relevant association factors of the previous model results, the interaction term analysis was employed to test the interaction between two significant variables. Statistics of the multilevel model were employed SPSS 21.0. (IBM, Armonk, NY, USA). A *P*-value less than 0.05 was considered as significant.

### Ethics

Not applicable as this is a secondary data analysis of a publicly available data set. Data set is distributable only by the 2015 China Migrant Dynamic Monitoring Survey and is available in the public domain through registration on the website http://www.chinaldrk.org.cn/wjw/#/application/index.

## RESULTS

### Utilization of essential public health services

**Figure 1:**
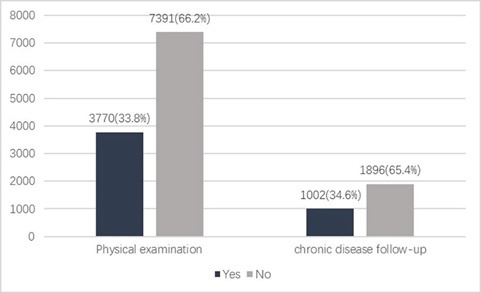
Essential public health services utilization among elderly migrants.

The study sample was comprised of 11161 old-aged migrants, and the utilization of EPHS of the participants is illustrated in **Figure 1**. Approximately66.2% of old-aged migrants did not access to the free annually physical examinations from community health station/center in the past year. Among the 2898 old-aged migrants with chronic diseases, only 34.6% of them have been follow-upped by doctors to monitor their conditions of chronic diseases in the past year of the investigated time.

### Differences in utilization of essential public health services

**Table 2** illustrated that differences in utilization of EPHS were found among different ages, ethnicity, exercise duration, migrating range and reason, number of local friends, monthly expenditure and income. Percentage of EPHS utilization was higher among those who were minorities, migrated cross-county, with more than 5 local friends, or from the family with less than ¥5000 montly income. In terms of the utilization of the free physical examination, the percentage was higher among those aged 70-80 years, in a generally-good physical health condition, with chronic diseases, with health insurance, or who migrated cross-county, exercised over 60 minutes, from the family monthly expended less than ¥2,000. For old-aged migrants with chronic diseases, the percentage was higher among those aged over 80 years, with more than 5 local friends, or who exercised 30-60 minutes, migrated for treatment, migrated cross-county, or from the family monthly expended ¥2,000-5000.

**Table 2:** Differences in utilization of EPHS among old-aged migrants with different variables, n (%)

Variable	Physical examinations		χ^2^	*P*-value	Follow-up services		χ^2^	*P*-value
**Yes**	**No**	**Yes**	**No**
**Age (years):**								
60-65					89 (30.5)	203 (69.5)		
65-70	1869 (32.2)	3933 (67.8)	22.647	<0.001	329 (30.4)	755 (69.6)	20.899	<0.001
70-80	1496 (36.6)	2594 (63.4)			412 (37.8)	677 (62.2)		
>80	405 (31.9)	864 (68.1)			172 (39.7)	261 (60.3)		
**Ethnicity:**								
Han	3351 (33.3)	6710 (66.7)	10.146	0.001	877 (33.5)	1744 (66.5)	15.072	<0.001
Else	419 (38.1)	681 (61.9)			125 (45.1)	152 (54.9)		
**Exercise:**								
<30 min	341 (23.4)	1115 (76.6)	122.032	<0.001	113 (29.3)	273 (70.7)	6.597	0.037
30-60 min	2054 (33.0)	4164 (67.0)			557 (36.2)	983 (63.8)		
>60 min	1373 (39.5)	2104 (60.5)			331 (34.2)	638 (65.8)		
**Range:**								
Cross-province	1262 (26.6)	3480 (73.4)	196.863	<0.001	381 (30.7)	860 (69.3)	17.456	<0.001
Cross-city	1353 (37.8)	2226 (62.2)			331 (35.7)	597 (64.3)		
Cross-county	1151 (40.6)	1683 (59.4)			290 (39.8)	439 (60.2)		
Cross-border	4 (66.7)	2 (33.3)			0 (0)	0 (0)		
**Reason:**								
Employment	732 (31.8)	1571 (68.2)	34.521	<0.001	150 (36.8)	258 (63.2)	25.425	<0.001
Caring children	1197 (31.9)	2559 (68.1)			298 (28.8)	737 (71.2)		
Treatment	21 (22.8)	71 (77.2)			21 (45.7)	425 (54.3)		
Retirement	1448 (37.1)	2454 (62.9)			445 (38.1)	724 (61.9)		
Else	327 (33.6)	736 (66.4)			88 (36.7)	152 (63.3)		
**Status:**								
Health	1656 (34.3)	3165 (65.7)	35.898	<0.001	234 (31.2)	517 (68.8)	7.190	0.066
General health	1740 (34.7)	3279 (65.3)			509 (34.9)	950 (65.1)		
Unhealth but self-care	346 (30.4)	793 (69.6)			227 (38.1)	369 (61.9)		
No self-care	28 (15.4)	154 (84.6)			509 (34.9)	950 (65.1)		
**Chronic disease:**								
Yes	929 (35.6)	1677 (64.4)	5.316	0.021	1002 (34.6)	1896 (65.4)		
No	2841 (33.2)	5714 (66.8)			0 (0)	0 (0)		
**Friends:**								
0	231 (17.7)	1074 (82.3)	243.886	<0.001	104 (25.3)	307 (74.7)	28.746	<0.001
1-5	1525 (31.7)	3281 (68.3)			415 (33.0)	841 (67.0)		
>5	2014 (39.9)	3036 (60.1)			483 (39.2)	748 (60.8)		
**Health insurance:**								
Yes	3313 (34.8)	6197 (65.2)	32.213	<0.001	889 (34.4)	1692 (65.6)	0.181	0.671
No	457 (27.7)	1194 (72.3)			113 (35.6)	204 (64.4)		
**Expenditure:**								
<¥2000	1043 (36.6)	1809 (63.4)	45.932	<0.001	208 (34.3)	398 (65.7)	18.000	<0.001
¥2000-5000	2356 (34.2)	4541 (65.8)			673 (36.8)	1158 (63.2)		
>¥5000	371 (26.3)	1041 (73.7)			121 (26.2)	340 (73.8)		
**Income:**								
<¥5000	1895 (35.6)	3426 (64.4)	43.924	<0.001	456 (36.4)	796 (63.6)	7.317	0.026
¥5000-10000	1593 (33.7)	3132 (66.3)			440 (34.4)	838 (65.6)		
>¥10000	282 (25.3)	833 (74.7)			106 (28.8)	262 (71.2)		

EPHS – essential public health service, ¥ – yuan.

### Factors associated with essential health services utilization

The estimation impact of different variables on the community physical examination utilization among old-aged migrants at the individual and household levels was shown in **Table 3**. Model 1shows the tested result of an empty model. The ICC was 0.909, and it indicated that ICC = 0.909, in other words, 9.1% of the total variation was caused by individual variation, while 90.9% of that was due to family variation. After adjusting for all factors in model 6, family and individual setting remained associated with the utilization of physical examinations for the old-aged migrants. It indicated that exercise time, reason for immigration, physical health condition, chronic disease, local friends, health insurance, expenditure, and income were associated with physical examination utilization. Old-aged migrants aged 70-80 years, in better health condition, with chronic disease, with health insurance, or gained less than ¥2000, or expended less than ¥5000 per month enjoyed a significantly sufficient utilization rate, while those whom exercised less time, with fewer local friends, or migrated for treatment had a significantly lower utilization rate.

**Table 3:** Multi-level model of family and individual settings associations with physical examinations utilization

Variable	β (SE)						
**Model 1**	**Model 2**	**Model 3**	**Model 4**	**Model 5**	**Model 6**	**Model 7**
**Fixed effects**							
**Intercept**	0.326 (0.005)‡	0.381 (0.019)‡	0.710 (0.229)†	0.586(0.230)*	0.598(0.228)†	0.529(0.229)*	0.702(0.282)*
**Age** (years, Ref: >80):							
65-70		0.022 (0.010)*	0.030 (0.011)†	0.024(0.011)*	0.016(0.011)	0.015(0.011)	0.014(0.011)
70-80		0.033 (0.010)†	0.039 (0.010)‡	0.035(0.010)‡	0.028(0.010)†	0.027(0.010)†	0.026(0.010)†
**Ethnicity (Han)**		-0.043 (0.017)*	-0.016 (0.017)	-0.018(0.017)	-0.020(0.017)	-0.017(0.017)	-0.018(0.017)
**Exercise** (Ref: >60)							
<30 min		-0.122 (0.012)‡	-0.118 (0.012)‡	-0.097(0.013)‡	-0.073(0.013)‡	-0.075(0.013)‡	0.020(0.039)
30-60 min		-0.041 (0.009)‡	-0.039 (0.009)‡	-0.035(0.009)‡	-0.025(0.009)†	-0.026(0.009)†	0.024(0.022)
**Range** (Ref: Cross-border)							
Cross-province			-0.425 (0.228)	-0.426(0.228)	-0.414(0.226)	-0.417(0.236)	-0.432(0.233)
Cross-city			-0.311 (0.228)	-0.309(0.228)	-0.306(0.226)	-0.318(0.226)	-0.335(0.230)
Cross-county			-0.278 (0.228)	-0.275(0.228)	-0.276(0.226)	-0.290(0.226)	-0.300(0.227)
**Reason** (Ref: Else):							
Employment			-0.015 (0.014)	-0.018(0.014)	-0.026(0.014)	-0.025(0.014)	-0.025(0.014)
Caring children			-0.018 (0.015)	-0.022(0.015)	-0.019(0.015)	-0.009(0.015)	-0.008(0.015)
Treatment			-0.136 (0.038)‡	-0.096(0.039)*	-0.091(0.038)*	-0.085(0.038)*	-0.080(0.038)*
Retirement			0.002 (0.015)	0.005(0.015)	0.007(0.015)	0.011(0.015)	0.010(0.015)
**Status** (Ref: No self-care)							
Health				0.133(0.023)‡	0.122(0.023)‡	0.124(0.023)‡	-0.159(0.067)*
General health				0.113(0.022)‡	0.104(0.022)‡	0.104(0.022)‡	-0.087(0.048)
Unhealth but self-care				0.080(0.022)‡	0.075(0.022)†	0.073(0.022)†	-0.019(0.031)
**Chronic disease** (Yes)				0.032(0.007)‡	0.032(0.007)‡	0.034(0.007)‡	0.032(0.007)‡
**Friends** (Ref: >5):							
0					-0.154(0.015)‡	-0.153(0.015)‡	-0.067(0.036)
1-5					-0.053(0.008)‡	-0.054(0.008)‡	-0.008(0.020)
**Health insurance**(Yes)					0.040(0.012)†	0.041(0.012)†	-0.036(0.027)
**Expenditure** (Ref: >5000 Yuan, ¥):							
<¥2000						0.051(0.023)*	0.050(0.023)*
¥2000-5000						0.034(0.019)	0.032(0.019)
**Income** (Ref: >10000¥)							
<¥5000						0.046(0.023)*	0.115(0.041)†
¥5000-10000						0.034(0.021)	0.062(0.025)*
**Range × Income**							0.022(0.011)*
**Exercise × Friends**							0.021(0.008)*
**Status × Range**							-0.022(0.006)‡
**Status × Insurance**							-0.045(0.014)†
**Random variance**							
**Residual**	0.020‡	0.020‡	0.020‡	0.020‡	0.020‡	0.020‡	0.020‡
**Intercept**	0.200‡	0.198‡	0.193‡	0.193‡	0.189‡	0.189‡	0.188‡
**-2log**	9317.641	9198.115	9020.216	8965.993	8832.784	8810.008	8776.531

*P < 0.05.

†*P* < 0.01.

‡*P* < 0.001.

By comparing the relevant indicators, model 7 was the optimal model. The interaction results (shown in **Table 3**) indicated that household income had significantly positive interaction with the migrating range, which means that income showed a significant moderating effect on the utilization among old-aged migrants with different migrating range. Local friends and exercise time also had a significant interaction on the utilization. While physical health condition showed significantly negative interaction with the migrating range and health insurance. It indicated that the migrating range and health insurance had increased the disparity of utilization among those with different physical health condition. Migrating range also increased the disparity among those with different monthly income.

**Table 4** shows the results of the multilevel model of the impact of different variables on the follow-up services utilization. The ICC was 0.987, indicating that 98.7% of the total variation was caused by family variation. Family and individual setting remained associated with the utilization of follow-up services for old-aged migrants. It indicated that ethic group, exercise time, migrating range, reason for migration, numbers of local friends, health insurance, expenditure were associated factors for follow-up services utilization. Old-aged migrants who exercised less than 30-60 minutes, expended less than ¥2000-5000 enjoyed a significantly higher utilization rate, while those who were Han group, with fewer local friends, exercised less than 30 minutes, migrated cross-province or for caring children had a significantly lower utilization rate.

**Table 4:** Multilevel model of family and individual setting associations with chronic follow-up services utilization

Variable	Β (SE)						
**Model 1**	**Model 2**	**Model 3**	**Model 4**	**Model 5**	**Model 6**	**Model 7**
**Fixed effects**							
**Intercept**	0.346 (0.010)‡	0.447 (0.033)‡	0.490 (0.041)‡	0.450 (0.045)‡	0.449 (0.048)‡	0.406 (0.058)‡	1.131 (0.272)‡
**Age **(years, Ref: >80)							
60-65		-0.002 (0.015)	0.007 (0.015)	0.007 (0.015)	-0.002 (0.015)	-0.002 (0.015)	0.007 (0.014)
65-70		0.006 (0.011)	0.016 (0.011)	0.015 (0.011)	0.007 (0.011)	0.007 (0.011)	0.013 (0.010)
70-80		0.003 (0.010)	0.009 (0.010)	0.009 (0.010)	0.002 (0.009)	0.002 (0.010)	0.005 (0.009)
**Ethnicity** (Han)		-0.118 (0.033)‡	-0.103 (0.033)†	-0.102 (0.033)†	-0.102 (0.033)†	-0.102 (0.033)†	-0.102 (0.033)†
**Exercise** (Ref: >60)							
<30min		-0.053 (0.015)‡	-0.059 (0.015)‡	-0.050 (0.015)†	-0.036 (0.015)*	-0.035 (0.015)*	-0.052 (0.014)‡
30-60 min		0.020 (0.011)	0.020 (0.011)	0.020 (0.011)	-0.023 (0.011)*	0.023 (0.011)*	0.022 (0.010)*
**Range** (Ref: Cross- county)							
Cross-province			-0.071 (0.024)†	-0.071 (0.024)†	-0.060 (0.025)*	-0.052 (0.025)*	-0.121 (0.037)†
Cross-city			-0.034 (0.026)	-0.034 (0.026)	-0.031 (0.026)	-0.028 (0.026)	-0.061 (0.029)*
**Reason** (Ref: Else)							
Employment			-0.018 (0.023)	-0.022 (0.023)	-0.033 (0.023)	-0.032 (0.023)	-0.028 (0.021)
Caring children			-0.060 (0.024)*	-0.063 (0.024)†	-0.056 (0.023)*	-0.053 (0.024)*	-0.052 (0.022)*
Treatment			0.019 (0.050)	0.015 (0.050)	0.023 (0.049)	0.023 (0.049)	0.032 (0.046)
Retirement			0.009 (0.024)	0.006 (0.024)	0.006 (0.024)	0.005 (0.024)	0.008 (0.022)
**Status** (Ref: No self-care):							
Health				0.032 (0.023)	0.024 (0.022)	0.026 (0.022)	-0.385 (0.101)‡
General health				0.048 (0.021)*	0.038 (0.020)	0.038 (0.020)	-0.241 (0.068)‡
Unhealth but self-care				0.044 (0.020)*	0.035 (0.020)	0.035 (0.020)	-0.117 (0.037)†
**Friends** (Ref: >5):							
0					-0.141 (0.022)‡	-0.142 (0.022)‡	-0.184 (0.055)†
1-5					-0.034 (0.010)‡	-0.034 (0.010)‡	-0.059 (0.026)*
**Health insurance** (Yes)					0.046 (0.020)*	0.045 (0.020)*	-0.096 (0.075)
**Expenditure** (Ref: >5000 Yuan, ¥)							
<¥2000						0.037 (0.044)	0.037 (0.044)
¥2000-5000						0.076 (0.034)*	0.077 (0.034)*
**Income** (Ref: >10000¥)							
<¥5000						-0.012 (0.042)	-0.013 (0.096)
¥5000-10000						-0.029 (0.037)	-0.032 (0.058)
**Status × Range**							-0.120 (0.019)‡
**Status × Friends**							-0.034 (0.011)†
**Status × Insurance**							0.068 (0.029)*
**Income × Friends**							0.014 (0.007)*
**Income × Insurance**							-0.016 (0.007) *
**Random variance**							
**Residual**	0.003‡	0.003‡	0.003‡	0.003‡	0.003‡	0.003‡	0.002‡
**Intercept**	0.223‡	0.222‡	0.219‡	0.219‡	0.219‡	0.218‡	0.218‡
**-2log**	2188.025	2145.096	2116.073	2109.612	2057.021	2049.504	1954.834

*P < 0.05.

†*P* < 0.01.

‡*P* < 0.001.

As the optimal model 7shown, physical health condition had significantly negative interaction with local friends and immigrating range, while it had positive interaction with health insurance. The migrating range and local friends have increased the disparity of utilization among the old-aged migrants with different physical health condition, while health insurance have decreased this disparity. Meanwhile, household income had significantly negative interaction with health insurance, while had significantly positive interaction and moderating effect on the service utilization among those with different numbers of local friends.

## DISCUSSION

In China, Hukou is an indispensable foundation for the management of population and delivering public services. In history, China's population management policy was implemented based on the Hukou system, which plays an important role in the seniors' refuge, retirement and relocation, and population migration, and links to diverse social security [16]. Although the Hukou Reform abolished the distinction between the agricultural system and non-agricultural system, the rights and benefits attached to these two systems are not completely equivalent. Many public services and social welfare policies have been directly linked to household registration which are difficult to be separated and it caused the discrimination against migrants. The reform did not change the fact that the migrants have limited access to these social entitlements, such as health care, employment, insurance, pension, and social support. Especially for the old-aged migrants, with the parallel of lacking of service delivering information and the proactive behavior in seeking health care, it also incurred the limited access to preventive and medical resources [17]. Generally, the cancellation of the agricultural account opened the prelude to the “era of Equal Rights”, but there is still a long way to achieve “equal rights” for the migrating population.

### Insufficient utilization of essential public health services

Utilization of EPHS for old-aged migrants was at a lower level (33.8%) when compared with the general population at the level of 43.3%. Only 34.6% of the old-aged migrants with chronic conditions received followed up services, which was less than half of those under the age of 15 years (71.3%) in the previous study [18]. Insufficient utilization may be associated with multiple factors such as wide migration range, weak health literacy, and policy awareness, poor family conditions, or that Hukou registration and social security system have not been fully connected, and the equalization of EPHS has not been achieved in this period.

Above all, migration was the core factor associated with the insufficient utilization. The larger the migrating range, the lower the service utilization rate, which is in accord with the results of previous research. In China, public policies linked to the household registration system. The special status of old-aged migrants makes it difficult for them to register in the immigrating area or receive the same social welfare as local residents [16]. Moreover, policies among different cities are fragmented. The senior migrating from rural to urban areas may be limited to participating in local social work projects [19]. The government should pay more attention to establish a connecting mechanism between the household registration system and social security system for the migrants. It also needs to improve the social welfare system for migrants and promote the adaptable local social and economic environment and public policies for old-aged migrants, clarify the responsibilities of various stakeholders [17].

### Family factors associated with the utilization

Service utilization of old-aged migrants was significantly different across different families. It indicated that the lower monthly household expenditure, the higher utilization. Household income had a positive reaction with local friends and migrating range. Household income has reduced the disparity of the utilization across individuals with different numbers of local friends, different migrating range. The evidence indicated that old-aged migrants were more likely to rely on family resources to seek health services was owing to the limited local social benefits [20,21]. Household economic factors could effectively stimulate the utilization of health services [22]. In hence, more attention should be paid to promoting the social interaction through families and improving old-aged migrants’ household and economic status.

Meanwhile, it shows that the elderly migrated for caring children were less likely to utilize EPHS, compared with the else. It might be related to family economic status and intergenerational conflicts [23]. Previous research found that the senior who care for generations are relatively vulnerable in family socioeconomic status [24]. To a certain extent, most old-aged migrants live with their children, and intergenerational conflicts require them to focus on their children and ignore themselves [25]. The government should provide equal or inclined development opportunities for senior workers. Besides, the government should eliminate inequitable policies and explore the establishment of a balanced, systematic floating population policy at the national level, covering production, work-related injuries.

### Heath status, friends associated with the utilization

Health status had a negative interaction with migrating range and number of local friends, increasing the disparity of the utilization among these populations. There were quantities of seniors migrating for treatment since the higher quality health resources are concentrated in modern cities [26]. This study found that the chronic conditions and longer excise were associated with the improvement of old aged migrants’ activeness in seeking EPHS. It could be explained by those who took regular exercise habits were generally concerned about their health [17], and they collect health information actively. Finally, it is easily satisfied with the expectation of the service in need. However, the lack of awareness of health needs is a serious problem among the majority of migrants [16]. As such, the services delivering system should strengthen health promotion project including health education and health literacy for the migrants [16]. The interventions could be carried out in areas where old-aged migrants gathered together or even required them participating in regular physical examinations [27]. Also, the staff should strengthen the early detection of common geriatric diseases and implement comprehensively follow-up management for migrants with chronic diseases in the delivering process [28].

The more local friends, the higher the public health services utilization rate. In this study, exercise time and local friends had a positive reaction and has decreased the difference in utilization. There is a tendency that the weaker the elderly migrants are, the fewer friends they have [5]. In accordance with previous findings in the general population [29]. As relevant research shows that elderly leaving from their hometown, they are isolated separated from society [30], then lose their original social capital and face communication barriers [26]. Simultaneously, due to the lack of ability to dealing with new things and social support [30,31], a sense of loneliness, strangeness, and rejection covers them and makes it is difficult to adapt to regional living habits [32]. As prior study [33] discovered that local friend accumulated social capital for migrants, and helps the senior to rebuild social networks and gain more emotional support [34]. The good social cohesion in the local area could improve the accessibility of EPHS for them [35]. Therefore, more attention should be paid to giving more social support by friends for old-aged migrants [36] and helping them be integrated into local life and find a sense of social belonging [37,38]. The local social organizations and community committees should play a role as liking bridge [39] between old-aged migrants and local residents, such as setting up interest groups or organizing more activities. Community committees and health institutions should improve the accessibility of the EPHS for the old-aged migrants with different backgrounds [40].

To the best of our knowledge, this study might be the first study focusing on the associated factors with the elderly migrants’ utilization of EPHS at the family and individual levels. Considerable disparity was found in the utilization of EPHS among old-aged migrants. The significant factors and their relationship might contribute to providing references for improving the utilization and social work. However, several limitations should not be ignored in this study. First, the data came from a cross-sectional survey, and it cannot determine the trends or long-term effects on migrants’ health service utilization. Second, retrospective self-assessment may lead to bias, and old-aged migrants may miss out some specific details during the investigating process. Third, how the selected variables specifically were associated with the utilization need to explore further.

## CONCLUSIONS

This study estimated the differences and underlying factors with the utilization of EPHS among old-aged migrants. The utilization of EPHS for old-aged migrants was insufficient when compared with the general population, and the migration was the main determinant. The lower economic household income, the weaker physical health condition, and fewer local friends were associated with the utilization of EPHS. This study might provide relevant references for the health issues of interregional migrants in other countries, preventive care provision, and healthy aging. In conclusion, the government should establish targeted special floating population policies and provide development opportunities for old aged workers to improve their social inclusion. The Health services delivering system should promote the equalization of EPHS for migrants and shape old-aged migrants’ healthy behavior in seeking public health services. Social organizations and the community should pay attention to old-aged migrants’ social support, and guide them adapt to the local life and rebuild social networks both in the household and individual level.
